# Macular ganglion cell complex layer thickness measured with spectral-domain OCT in a large population-based cohort study

**DOI:** 10.1186/s12886-025-03989-x

**Published:** 2025-04-16

**Authors:** Caixia Lin, Lixia Ma, Aiguo Lv, Hongyi Liu, Qing Pan, Kai Cao, Xu Jia, Sujie Fan, Jian Wu, Ningli Wang

**Affiliations:** 1 Beijing Institute of Ophthalmology, Beijing Tongren Eye Center, Beijing Tongren Hospital, Beijing Key Laboratory of Intelligent Diagnosis Technology and Equipment for Optic Nerve-Related Eye Diseases, Capital Medical University, Beijing, 100730 China; 2 Henan Academy of Innovations in Medical Science, Zhengzhou Aviation Port Economic Zone, No. 1, Bio-Technology Street, Henan, 450008 China; 3 Handan City Eye Hospital, Handan, 056001 China; 4 Department of Epidemiology and Biostatistics, West China, School of Public Health and West China Fourth Hospitalaq, Sichuan University, Chengdu, Sichuan 610041 P. R. China; 5 State Key Laboratory of Ophthalmology, Guangdong Provincial Key Laboratory of Ophthalmology and Visual Science, Zhongshan Ophthalmic Center, Sun Yat-Sen University, Guangzhou, 510060 China; 6 The First Affiliated Hospital (College of Clinical Medical), Henan University of Science and Technology, Luoyang, 471000 China

**Keywords:** GCC thickness, SD-OCT, The Handan Eye Study

## Abstract

**Purpose:**

To establish the normal GCC thickness profile in the general population using SD-OCT in different macular sectors. Determining the systemic and ophthalmic factors associated with GCC thickness and further identifying the potential risk factors were the secondary objectives.

**Methods:**

Participants in the population-based cohort study had to be at least thirty years old. Every participant had a routine ophthalmological examination. Using SD-OCT, the GCC thickness was determined. To assess the relationship between GCC thickness and systemic and ocular characteristics, mixed linear models were used. R V.4.1.1 was the statistical analysis program utilized.

**Results:**

Two thousand four hundred ninety subjects, average age 56.60 ± 10.39 years, were collected in this analysis. GCC average thickness measured was 95.57 ± 7.47 μm. The GCC thickness of the superior (95.46 ± 7.87 μm) was the thinnest, and the inferior subfield (95.68 ± 7.66 μm) was thickest. In the multivariate regression analysis, a thinner GCC was majorly linked to being older (*P* < 0.001), current smoking (*P* < 0.001), no diabetes (*P* = 0.014), and a larger vertical cup disc ratio (VCDR) (*P* < 0.001). When the data was divided by age, a thinner GCC was also connected to female (*P* < 0.001), current smoking (*P* = 0.006), having high systolic blood pressure (SBP) (*P* = 0.002), lower low-density lipoprotein (LDL) (*P* = 0.002), higher triglycerides (TG) (*P* = 0.029), higher best corrected visual acuity (BCVA) (*P* < 0.001), and a larger vertical cup disc ratio (VCDR) (*P* = 0.002). Stratified by axial length (AL), a thinner GCC was associated with advanced age (*P* < 0.001), female (*P* = 0.005), and a larger VCDR (*P* = 0.001).

**Conclusions:**

Our study provides benchmarks for GCC thickness, distribution, and linked ocular and systemic factors among middle-aged individuals in rural China, emphasizing the strong correlation between GCC thickness and multiple ocular and systemic variables. Additionally, our findings underscore the importance of establishing global normative databases to address ethnic variations in GCC thickness and the distinctiveness of related ocular and systemic factors.

**Supplementary Information:**

The online version contains supplementary material available at 10.1186/s12886-025-03989-x.

## Introduction

Around 8.4 million individuals worldwide suffer from bilateral blindness due to glaucoma, which is one of the main causes of blindness and affects up to 3% of the population over 40 [1, 2]. Glaucoma is characterized by progressive degeneration of retinal ganglion cells (RGC) and the axons, which results in thinning of the retinal nerve fiber layers (RNFL) and irreversible visual field (VF) defects [3–5]. The VF is considered the gold standard clinical test for glaucoma diagnosis [6]. Several studies have investigated that RGCs may lose prior to detectable VF defect; therefore, the sensitive and accurate measurement of RGCs was extremely indeed [7–9]. The RNFL, the ganglion cell layer (GCL), and the inner plexiform layer (IPL) made up the macular ganglion cell complex (GCC). Since numerous RGCs are within the macula section, the evaluations of GCC are beneficial for glaucoma early detection [10–12].

In recent years, spectral-domain OCT (SD-OCT) has been widely used in both scientific research and clinical practice, with high spatial resolution and acquisition speed in a non-invasive way [13–15]. The automated segmentation and measuring of specific retinal layers are becoming increasingly possible using SD-OCT technology. The assessment of GCC thickness has been widely used in the diagnosis and monitoring of glaucoma.

As the SD-OCT devices have evolved over time, quite a few changes have been made in OCT regarding reference databases. Previous studies have conducted the parameters are different among each ethnic population [16], yet most normative databases used in SD-OCT software are obtained in Caucasians and limited by a low representation of Asian subjects. Meanwhile, previous studies are usually derived from hospital-based or small sample populations, giving rise to potential selection bias. In addition, high-myopic individuals may also have the relevant optic disc structural changes, such as deformation and thinner pre-laminar tissue, which is like glaucoma; therefore, well-described RGC-specific assessment parameters will be essential to assist clinical diagnosis.

In the present study, the primary objective was to establish the normal GCC thickness profile in the general population using SD-OCT in different macular sectors. Determining the systemic and ophthalmic factors associated with GCC thickness and further identifying the potential risk factors was the secondary objective.

## Methods

### Study population

A population-based cohort study, the Handan Eye Study (HES), involved participants in Handan, Hebei Province, northern China, who were at least thirty years of age. The detailed methodologies of HES have been illustrated elsewhere [17]. Briefly, 6830 individuals (response rate: 90.4%) took part in HES at baseline phase in 2006–2007. While 5394 subjects (response rate: 85.3%) of the survivors participated in the follow-up study during 2012–2013. As the OCT was not conducted in the baseline visits, the data used in this study was from the follow-up visits. The study protocol was approved by the Beijing Tongren Hospital Ethics Committee (TREC2006-22), and written informed consent was obtained from all the participants, according to the Declaration of Helsinki.

Meanwhile, we exclude individuals with glaucoma (i.e., optic disc with hemorrhage, thinning retinal nerve fiber layer thickness, and any glaucomatous visual field defects) or intraocular pressure (IOP) > 21 mmHg, retinal disease (like diabetic retinopathy or diabetic macular edema), and other vitreoretinal diseases. Eyes having pathologic retinal lesions or spherical equivalents (SEs) less than −8.0 diopter (D) were further disqualified to further separate out the eyes with pathologic myopia. As a result, a total of 4548 eyes from 2552 patients were enrolled in this study.

### Systemic examination

All participants underwent a standard systematic and ocular examination by well-trained and experienced ophthalmologists. Information on demographics, educational backgrounds, and medical histories (including smoking, drinking, diabetes, hypertension, and known significant systemic disorders) were gathered through the questionnaire. Weight divided by height was used to compute the body mass index, or BMI. Blood was drawn for biochemical analysis, which included measurements of blood urea nitrogen, serum creatinine, fasting glucose, and lipid indicators such as total cholesterol, total TG, LDL and HDL.

### Ocular examination

The obedient participants had slit-lamp biomicroscopic (Topcon SL-2F; www.global.topcon.com) and had their IOP monitored using a Kowa applanation tonometer HA-2 (Kowa Company Ltd. Tokyo, Japan). Visual acuity was tested monocularly and then binocularly at 4 m using a log MAR chart. AL was examined with a 10 MHz A/B-mode ultrasound device (Cine Scan, Quantel Medical, Clermont-Ferrand, France). The refraction and corneal curvature were measured using an autorefractor (KR8800, Topcon, Tokyo, Japan) without pupil dilation.

### OCT measurement procedures

A skilled operator performed GCC thickness scans with a spectral-domain OCT (RTVue 100–2, Optovue, Inc., Fremont, CA; version 4.0). The device used a near-infrared light source with a wavelength of 840 nm and obtained 26,000 A-scans per second. The optic disc was manually positioned in the middle of the scanned image, with 12 radial scans (ranging from 1.3 to 4.9 mm) and 6 concentric rings. The scan imaging covered a measurement area of 3.4 mm in diameter ring that included the optic nerve head and surroundings. The single scan with the best quality was selected from the 3-time scan repeats for each eye analysis. The completed cross-sectional images were acquired in individual radial scans, and without artifacts (boundary errors or decentration), they were acceptable. All GCC model images were manually checked to ensure that the optic nerve head was evident in the center of the scan. Various optic nerve head parameters were measured by algorithms native to RTVue OCT automatically. Results were considered credible if SSI (signal strength index) ≥ 45. Moreover, one glaucoma specialist reviewed those scans carefully for abnormalities. Those that did not meet the criteria were excluded further from the analysis.

### Statistical methods

#### Statistics (macular paper)

The statistical analysis was carried out using the R software (version 4.1.1; R Core Team, 2021). Descriptive statistics were utilized to describe the demographic, RT, and other characteristics of the included and excluding groups. The normality distribution and variance homogeneity of continuous variables were assessed, and the t-test or Wilcoxon rank sum test was used. For ordered data, the chi-square test was used, whereas the non-parametric Wilcoxon rank sum test was used for disordered data. The relationships between RT (central macular subfield, average inner ring, and average outer ring) and biochemical traits were then examined using univariate and multivariate linear regression models. First, we used a single-factor mixed-effects regression model to estimate the relationship between behavioral traits, medical history, central macular thickness, inner and outer ring measurements, and ocular parameters. Secondly, variables that were clinically important or statistically significant (*P* ≤ 0.1) were included in the multi-factor mixed-effects regression model. The all-subset selection procedure was then used to select the most relevant variables. *P* values < 0.05 were considered statistically significant.

## Results

### Comparisons of demographic and biochemical characteristics between female and male

During the Handan eye study follow-up period, 4793 of 5394 underwent the SD-OCT examination. The demographic features of the research population were described in Table 1. In total, 2490 subjects with healthy and high-quality imaging incorporated into the analysis (1379 men and 1111 women). The average age was 56.60 ± 10.39 years, with a range of ages from 35 to 86. Compared the demographic parameters in two groups, the male tended to be significantly higher age (*P* < 0.001), higher level of education (*P* < 0.001), current smoking (*P* < 0.001), lower BMI (*P* < 0.001), less hypertension (*P* = 0.011), lower lipids (*P* < 0.001), less diabetes (*P* < 0.001), lower keratometry (*P* < 0.001), lower SE (*P* < 0.001), lower BCVA (*P* < 0.001), lower IOP (*P* < 0.001), longer AL (*P* < 0.001), deeper Anterior chamber depth (ACD) (*P* < 0.001), enlarged VCDR (*P* < 0.001). The differences in other features between the two groups are given in Table 1.

**Table 1:** Gender differences in demographics and biochemistry

**Characteristic**	**Overall** *N* = 2,490^a^	**Female** *N* = 1,379^a^	**Male** *N* = 1,111^a^	***p*-value**^b^
Age (years)	56.60 (10.39)	55.89 (10.09)	57.48 (10.68)	< 0.001
Education				< 0.001
Below high school	2,400 (96%)	1,355 (98%)	1,045 (94%)	
High school or above	90 (3.6%)	24 (1.7%)	66 (5.9%)	
Current smoker				< 0.001
Yes	637 (26%)	1 (< 0.1%)	636 (58%)	
No	1,820 (74%)	1,360 (100%)	460 (42%)	
Weight (kg)	64.57 (11.01)	61.79 (10.11)	68.02 (11.12)	< 0.001
Height (cm)	159.36 (8.00)	154.31 (5.52)	165.62 (5.89)	< 0.001
BMI (kg/m^2^)	25.41 (3.75)	25.93 (3.84)	24.77 (3.54)	< 0.001
Hypertension				0.011
Yes	709 (30%)	421 (32%)	288 (27%)	
No	1,673 (70%)	899 (68%)	774 (73%)	
SBP (mmHg)	140.05 (21.22)	138.76 (21.37)	141.65 (20.93)	< 0.001
DBP (mmHg)	81.59 (12.11)	80.53 (11.40)	82.91 (12.82)	< 0.001
CHD				0.150
Yes	204 (8.8%)	122 (9.6%)	82 (7.9%)	
No	2,110 (91%)	1,150 (90%)	960 (92%)	
TG (mmol/L)	1.35 (1.02)	1.38 (0.90)	1.32 (1.15)	< 0.001
HDL (mmol/L)	1.24 (0.28)	1.28 (0.28)	1.18 (0.26)	< 0.001
LDL (mmol/L)	2.60 (0.72)	2.67 (0.73)	2.52 (0.69)	< 0.001
Diabetes				< 0.001
Yes	85 (3.6%)	65 (5.0%)	20 (1.9%)	
No	2,256 (96%)	1,231 (95%)	1,025 (98%)	
HbA1c (%)	5.73 (0.73)	5.76 (0.81)	5.70 (0.61)	0.700
Keratometry OD (D)	44.20 (1.53)	44.51 (1.48)	43.80 (1.49)	< 0.001
Keratometry OS (D)	44.19 (1.54)	44.53 (1.50)	43.77 (1.49)	< 0.001
SE OD (D)	0.28 (1.70)	0.30 (1.83)	0.26 (1.50)	0.089
SE OS (D)	0.33 (1.74)	0.38 (1.71)	0.26 (1.76)	0.062
BCVA OD (logMAR)	0.59 (0.26)	0.61 (0.26)	0.56 (0.25)	< 0.001
BCVA OS (logMAR)	0.57 (0.26)	0.59 (0.26)	0.55 (0.26)	0.004
IOP OD (mmHg)	11.35 (2.40)	11.41 (2.36)	11.28 (2.45)	0.120
IOP OS (mmHg)	11.96 (2.44)	12.06 (2.37)	11.83 (2.52)	0.007
CCT OD (μm)	530.59 (30.12)	530.17 (29.87)	531.11 (30.43)	0.200
CCT OS (μm)	530.51 (30.11)	529.86 (30.34)	531.32 (29.82)	0.200
AL OD (mm)	22.81 (1.08)	22.67 (1.19)	22.97 (0.90)	< 0.001
AL OS (mm)	22.76 (0.85)	22.57 (0.86)	23.00 (0.79)	< 0.001
ACD OD (mm)	2.78 (0.37)	2.74 (0.36)	2.82 (0.38)	< 0.001
ACD OS (mm)	2.77 (0.36)	2.72 (0.35)	2.83 (0.37)	< 0.001
VCDR OD	0.35 (0.13)	0.34 (0.13)	0.36 (0.13)	< 0.001
VCDR OS	0.34 (0.13)	0.33 (0.13)	0.35 (0.13)	0.003

*BMI* Body mass index, *SBP* Systolic blood pressure, *DBP* Diastolic blood pressure, *CHD* Coronary heart disease, *TG* Triglycerides, *HDL* High-density lipoprotein, *LDL* Low-density lipoprotein, *SE* Spherical equivalent, *BCVA* Best corrected visual acuity, *IOP* Intraocular pressure, *CCT* Central corneal thickness, *AL* Axial length, *ACD* Anterior chamber depth, *VCDR* Vertical cup disc ratio, *D* Diopter

^a^Mean (SD); n (%)

^b^Wilcoxon rank sum test; Pearson's Chi-squared test; Fisher’s exact test

### Distribution of GCC thickness in healthy eyes

Table 2 displayed the normative profile of overall, superior, and inferior subfields in 1%, 5%, 50%, 95%, and 99% percentiles. All parameters were represented as mean ± standard deviation (M ± SD). It was evident that the GCC thickness of the superior (95.46 ± 7.87 μm) was the thinnest, followed by the average thickness (95.57 ± 7.47 μm), and that of the inferior subfield (95.68 ± 7.66 μm) was thickest.

**Table 2:** Distribution of ganglion cell complex thickness in healthy eyes

Variables	Mean	SD	Q0.01	Q0.05	Q0.5	Q0.95	Q0.99
GCC Average Thickness (μm)	95.57	7.47	76.71	83.59	95.55	107.28	111.92
GCC Thickness Inferior (μm)	95.68	7.66	76.22	83.16	95.85	107.66	112.92
GCC Thickness Superior (μm)	95.46	7.87	75.03	83.00	95.65	107.52	112.82

*SD* standard deviation, Q0.01: 1% percentile; Q0.05: 5% percentile; Q0.5: 50% percentile; Q0.95: 95% percentile; Q0.99: 99% percentile; GCC stands for ganglion cell complex

### The correlation between gender and age in GCC

GCC thickness was expressed by the mean and standard deviation in each age group, and the changing trend with age for each measurement was significant for each measurement (Table 3).

**Table 3:** Distribution of ganglion cell complex thickness in healthy eyes by age

Characteristic	< 40 (*N* = 330)	41—50 (*N* = 1091)	51—60 (*N* = 1655)	61—70 (*N* = 1384)	≥ 70 (*N* = 473)	*F*-value	*P*-value
GCC Average Thickness (μm)	98.27 ± 0.54	97.65 ± 0.61	96.45 ± 0.59	94.49 ± 0.60	92.03 ± 0.69	41.631	** < 0.001**
GCC Thickness Inferior (μm)	98.38 ± 0.55	97.76 ± 0.63	96.54 ± 0.60	94.66 ± 0.61	92.23 ± 0.71	37.734	** < 0.001**
GCC Thickness Superior (μm)	98.17 ± 0.56	97.56 ± 0.64	96.38 ± 0.61	94.31 ± 0.62	91.82 ± 0.72	40.166	** < 0.001**

*SE* Standard error, *GCC* Stands for ganglion cell complex

**Fig. 1:**
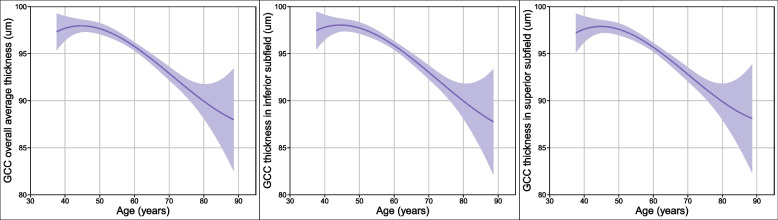
GCC thickness changes overall, inferior subfield and superior subfield along with age. The smooth curves in the figure represent the average GCC thickness in three subfields and the purple areas represent the 95% GCC thickness confidence interval

Five age groups were created from individuals: under 39, 40 to 49, 50 to 59, 60 to 69, and over 70 years. All GCC thickness subfields were thickest in the under-39 age group and thinnest in the over-70 age group. Figure 1 depicts a general tendency toward thinner GCC thickness as age increases.

The tendency for sex variation was demonstrated in Table 4. Compared with males, females had significantly thinner GCC thickness in the inferior subfield (*P* < 0.001).

**Table 4:** Distribution of ganglion cell complex thickness in healthy eyes by gender

Characteristic	Female (*N* = 2,744)	Male (*N* = 2,189)	t	*P*-value
GCC Average Thickness (μm)	95.56 ± 0.19	96.20 ± 0.29	2.185	** < 0.05**
GCC Thickness Inferior (μm)	95.53 ± 0.20	96.55 ± 0.29	3.487	** < 0.001**
GCC Thickness Superior (μm)	95.63 ± 0.20	95.86 ± 0.36	0.749	0.454

*SE* Standard error, *GCC* Stands for ganglion cell complex

### Average GCC thickness and its relationship to demographic and ocular characteristics

To estimate the independent relationships between demographic and ocular factors and average GCC thickness, univariate and multivariate regression models were employed (Table 5). The result demonstrated that thinner GCC thickness was substantially associated with older age (*P* < 0.001), female (*P* = 0.038), limited education (*P* < 0.001), current smoking (*P* = 0.006), higher SBP (*P* < 0.001) higher LDL (*P* = 0.015), without diabetes (*P* < 0.001), higher HbA1c (*P* = 0.007), and better VCDR (*P* < 0.001). In the multivariate model, the variables that were determined to be statistically significant in the univariate model or factors having clinical value were further included. Results from the multivariate model demonstrated that a thinner GCC was majorly linked to being older (*P* < 0.001), current smoking (*P* < 0.001), no Diabetes (*P* = 0.014), and a larger vertical cup disc ratio (VCDR) (*P* < 0.001) (Table 5). Generally, among systemic factors, age appears to have the strongest association with GCC thickness, while AL is most influential among ophthalmic factors. Accordingly, we performed univariate and multivariate analyses stratified by age and AL to account for potential confounding effects. When the data was divided by age, mean GCC thickness was also connected to female (*P* < 0.001), smoking (*P* = 0.006), SBP (*P* = 0.002), LDL (*P* = 0.002), TG (*P* = 0.029), BCVA (*P* < 0.001), and VCDR (*P* = 0.002) (Table S2). Stratified by AL, mean GCC thickness was associated with age (*P* < 0.001), female (*P* = 0.005), and VCDR (*P* = 0.001) (Table S3).

**Table 5:** The regression models for the relationship between demographic and biochemical characteristics and average GCC thickness

Variables	Univariate regression model				Multivariate regression model			
	**B**	**SE**	***t***	***P-value***	**B**	**SE**	***t***	***P-value***
Age (years)	−0.20	0.02	−10.84	** < 0.001**	−0.20	0.02	−10.89	** < 0.001**
Gender	0.71	0.34	2. 08	**0.038**				
Education	0.87	0.20	4.29	** < 0.001**				
Current smoker	−1.06	0.38	−2.77	**0.006**	−1.33	0.37	−3.58	** < 0.001**
BMI (kg/m^2^)	−0.02	0.05	−0.36	0.720				
SBP (mm Hg)	−0.05	0.01	−5.81	** < 0.001**				
DBP (mm Hg)	−0.03	0.01	−1.88	0.061				
CHD	1.05	0.59	1.8	0.073				
TG (mmol/L)	−0.30	0.16	−1.86	0.064				
HDL (mmol/L)	0.65	0.63	1.04	0.300				
LDL (mmol/L)	−0.57	0.23	−2.43	**0.015**				
Diabetes	3.03	0.89	3.42	** < 0.001**	2.10	0.86	2.45	**0.014**
HbA1c (%)	−0.61	0.22	−2.72	**0.007**				
Keratometry (D)	−0.61	0.10	−1.56	0.120				
SE (D)	−0.04	0.09	−0.51	0.610				
BCVA (logMAR)	−3.39	0.57	−5.97	0.120				
IOP (mmHg)	−0.05	0.05	−0.99	0.320				
CCT (μm)	0.00	0.00	0.66	0.510				
AL (mm)	−0.12	0.10	−1.13	0.260				
ACD (mm)	0.12	0.32	0.38	0.710				
VCDR	−4.15	1.11	−3.73	** < 0.001**	−4.79	1.08	−4.42	** < 0.001**

Gender: male VS. female; Education: below high school VS. high school or above

**Fig. 2:**
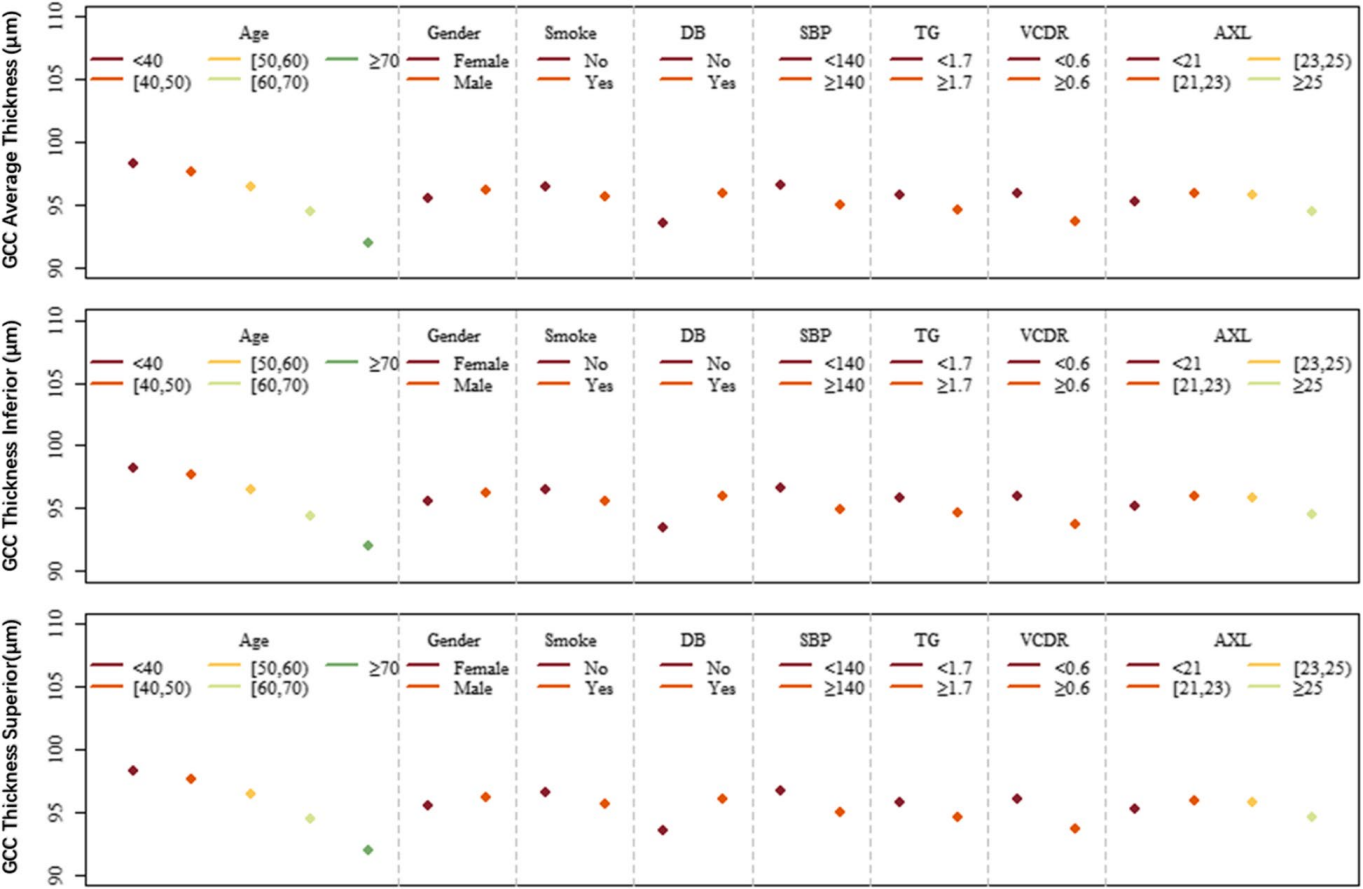
The relationship between demographic characteristics, medical history, behavior, ocular parameters, and GCC thickness using multivariate linear regression analysis in overall, inferior, and superior subfields

Figure 2 has intuitively shown the relationships of these parameters. GCC thickness decreased with age, while male subjects had thicker GCC thickness than females. Subjects who smoked did not have diabetes; lower HbA1c had thicker GCC thickness than others. However, education and HbA1c also had an inconsistent effect on GCC thickness, and the details were shown in Fig. 2.

## Discussion

We evaluated the GCC thickness and demonstrated normative profile distribution by SD-OCT in a Chinese rural adult population. The results demonstrated that the GCC thickness in the inferior subfield was the thickness, followed by the average GCC thickness, and the superior was the thinnest. In addition, the GCC thickness thinned with age, while other factors such as gender, smoking, diabetes, HbA1c, SBP, TG, LDL, BCVA, and VCDR were demonstrated to be connected to the thickness of the GCC.

In this study, the average GCC thickness measured 95.57 ± 7.47 μm, with the lower and upper subfields at 95.68 ± 7.66 μm and 95.46 ± 7.87 μm respectively. These values were slightly thicker than those reported in Chansangpetch S’s [16] study (91.25 μm, 90.82 μm, 91.70 μm) but thinner compared to other previous studies [18, 19] and the normative database, which lists an average GCC thickness of 105.3 ± 7.0 μm for 3D-OCT and 98.28 ± 9.31 μm for RTVue (Table S1).The variations may result from the various SD-OCT equipment [20], as well as variations in the demographics of the subjects, ethnic groups, and study inclusion criteria. Measurements from different OCT devices are not transferable [21], due to SD-OCT instruments using a different segmentation algorithm of the posterior boundary for measuring GCC thickness, and every OCT evaluates measures of several retinal total surfaces.

Previous studies have revealed that age is a significant factor influencing the distribution of GCC thickness. Bloch et al. also indicated that the average GCC thickness and all quadrants dropped considerably with age [18]. The reason may be caused by the RNFL thickness decreasing by 2–4 μm per ten years of age in individuals over 50 years, while the histological analysis also discovered that age-related RNFL thinning [22]. The explanation for how aging affects the RNFL layer could be related to the fundus's declining blood supply and the optic nerve's senescence apoptosis as aging advances [23]. Furthermore, as age progresses, systemic factors influencing GCC thickness assessment should not be ignored. To improve the accuracy of GCC thickness measurement, it is essential to synthesize data from numerous studies and determine a pattern of GCC thickness degradation with age. However, GCC thickness for different age groups may need to be taken into consideration independently.

Different from the results of the UK Biobank study [24], there was no discernible variation in the average GCC thickness between males (95.79 ± 7.58) and females (95.39 ± 7.38), which was similar to the results that no gender differences were found in the GCC's mean thickness in a Chinese sample as well [25] and another study conducted in Europeans [18]. However, after conducting a multivariate analysis stratified by age and AL, we found that gender influences the average thickness of the GCC. Similar to a study conducted in animal models, because females may have a higher ratio of parvocellular to magnocellular retina ganglion cells than males do, the study showed that females have thinner retinas than males [26].

For systematic parameters, multiple studies have suggested that hypertension was linked with GCC thinning, which may point to a crucial concern when utilizing OCT measures to make diagnoses [18, 27–29]. Therefore, we investigated the relationship between GCC thickness and systolic and diastolic blood pressure. The results have shown that GCC thickness is thinner in individuals with SBP in the multivariate analysis.

There were also varieties of studies that have displayed that diabetes may affect the retina and lead to neurodegenerative disease, with thinning GCC and RNFL thickness [30–34]. Thangamathesvaran et al. [30] revealed that GCC loss was observed in people with diabetes for ten to twenty years. This suggested that ganglion cell loss may occur prior to the development of diabetic retinopathy, indicating the beginning of neuronal death prior to any alterations in the vasculature. We did find a correlation between a thinner GCC and HbA1c (*P* = 0.049) and increasing TG (*P* < 0.001), which was consistent with the previous study [35].

Other than age, gender, and systematic parameters, it was found in the multivariate regression analysis that other ocular factors were associated with GCC thickness. GCC increased with the decreased keratometry, BCVA and VCDR; the reason might be this study hasn’t included the ocular magnification in the analysis, which compounding elements have a crucial effect on the GCC thickness and how it relates to related factors. The data included in our study was not corrected prior to the picture collection of the 3000 participants since the Optovue-RTVue100 instrument was not intended to fix the magnification after imaging [36]. However, the pathogenesis of glaucoma is diverse and remains unclear to this day [37, 38]. Meanwhile, in line with earlier research, we did not find a correlation between IOP and GCC thickness in this investigation [18, 39, 40]. Although IOP beyond threshold will harm ganglion cells at the lamina cribrosa [18], there is growing evidence for the role of the translaminar cribrosa pressure differential (TLCPD, IOP minus cerebrospinal fluid pressure), and the relationship between IOP and intracranial may be associated with the pathogenesis of glaucoma [41].

Since normative profiles from several manufacturers are derived from samples that contained low proportions of Asian individuals, the RTVue, Cirrus, and Spectralis databases have 22%, 24%, and 7% Asians, respectively. Thus, the Chinese people may receive incorrect diagnoses because of the built-in deviation plot. In our study, we presented the distribution, including mean and standard deviation, 1, 5, 50, 95, and 99 percentiles of GCC thickness, and may assist ophthalmologists in understanding the racial differences. However, compared to a mixed-racial normative database, the accuracy of employing a race-specific normative database is still unclear, necessitating more research.

In contrast to earlier hospital-based research, the selection bias was reduced in the population-based HES study. Furthermore, the association of GCC thickness with a variety of systemic and ocular factors in a standardized pattern was analyzed. However, there were still some limitations to our study. Firstly, as our study was cross-sectional and population-based, it was not possible to determine a causal relationship between the characteristics examined and the GCC; therefore, we were unable to make any inferences. Secondly, although this study excluded the abnormal eyes with possible pathology on OCT, the inclusion of the individuals who underwent cataract surgery may be the potential confounders for the results. Thirdly, about 40% of the participants in our study were eliminated in the final analysis because of the rigorous inclusion criteria, which only comprised healthy eyes, which may lead to potential susceptibility to missing data. Thus, there may be limitations to the generalizability of the findings of our investigation.

In conclusion, our study provides normative profile data of GCC thickness, distribution patterns, and correlated systemic and ocular parameters in a middle-aged cohort of rural population in China. We demonstrated the association between GCC thickness and gender, smoking, diabetes, HbA1c, SBP, TG, LDL, BCVA, and VCDR. When using GCC to make clinical judgments about glaucoma and other visual neuropathies marked by the loss of retinal ganglion cells, age is a significant factor to consider. GCC thickness was not shown to be a characteristic that was independently correlated with BMI, IOP, and AL. To learn more about the associated components that underline GCC thickness, more research is needed.

## Supplementary Information

Supplementary Material 1.

## Data Availability

All data generated or analyzed during this study are included in this article. Further inquiries can be directed to the corresponding author.
