# Supplementary material for: Macular ganglion cell complex layer thickness measured with spectral-domain OCT in a large population-based cohort study
Source: BMC Ophthalmol. 2025 Apr 16;25:214. doi: 10.1186/s12886-025-03989-x (PMC12001471; doi:10.1186/s12886-025-03989-x)
Supplement: Supplementary file 1 — Supplementary Material 1. [file 12886_2025_3989_MOESM1_ESM.docx]

| Supplementary Table 1.: Comparison with normative databases. | |
| --- | --- |
| **Average thickness of GCC (um)** | **Manufacturer** |
| 105.3±7.0 | 3D-OCT device (Topcon, Tokyo,Japan) |
| 98.28±9.31 | Optovue RTVue |
| 95.57±7.47 | Handan Eye Study |

Supplementary Table 2. Demographic and biochemical factors in GCC thickness by age groups.

**Univariate regression model**

| **Characteristic** | **≤40 years** | | | **41 - 49 years** | | | **50 - 59 years** | | | | **60 - 69 years** | | | | **≥ 70 years** | | |
| --- | --- | --- | --- | --- | --- | --- | --- | --- | --- | --- | --- | --- | --- | --- | --- | --- | --- |
|  | **B** | **SE** | **P-value** | **B** | **SE** | **P-value** | **B** | **SE** | **P-value** | **B** | | **SE** | **P-value** | **B** | | **SE** | **P-value** |
| Gender | 1.76 | 2.79 | 0.25 | 1.96 | 0.65 | **0.003** | 0.36 | 0.53 | 0.50 | 1.55 | | 0.64 | **0.02** | 0.07 | | 1.13 | 0.95 |
| Education | 2.47 | 1.85 | 0.20 | 0.33 | 0.47 | 0.49 | 0.46 | 0.34 | 0.18 | 0.21 | | 0.39 | 0.59 | 0.22 | | 0.53 | 0.68 |
| Current smoker | 0.00 | 3.50 | 0.00 | -1.70 | 0.77 | **0.028** | -0.51 | 0.58 | 0.38 | -2.18 | | 0.72 | **0.003** | -0.60 | | 1.19 | 0.62 |
| BMI (kg/m2) | 0.01 | 0.41 | 0.98 | -0.06 | 0.09 | 0.52 | -0.03 | 0.07 | 0.62 | -0.06 | | 0.09 | 0.47 | -0.11 | | 0.15 | 0.47 |
| SBP (mmHg) | 0.00 | 0.10 | 0.97 | -0.05 | 0.02 | **0.01** | -0.02 | 0.01 | 0.11 | -0.01 | | 0.02 | 0.40 | -0.04 | | 0.02 | 0.10 |
| DBP (mmHg) | -0.04 | 0.11 | 0.76 | -0.06 | 0.02 | **0.01** | -0.02 | 0.02 | 0.30 | -0.04 | | 0.03 | 0.16 | -0.08 | | 0.05 | 0.09 |
| Diabetes | 4.87 | 4.68 | 0.31 | -1.13 | 4.30 | 0.79 | 2.15 | 1.49 | 0.15 | 2.49 | | 1.28 | 0.05 | 3.02 | | 3.13 | 0.34 |
| HbA1c (%) | 0.65 | 1.80 | 0.72 | 0.15 | 0.55 | 0.78 | -0.53 | 0.36 | 0.13 | -0.48 | | 0.38 | 0.21 | -0.15 | | 0.66 | 0.82 |
| HDL (mmol/L) | 2.14 | 5.08 | 0.68 | -0.54 | 1.19 | 0.65 | 0.73 | 0.96 | 0.45 | 1.09 | | 1.21 | 0.37 | 0.60 | | 1.99 | 0.77 |
| LDL (mmol/L) | 3.92 | 1.21 | **0.004** | -0.15 | 0.46 | 0.75 | -0.25 | 0.37 | 0.49 | -0.55 | | 0.45 | 0.23 | -1.72 | | 0.70 | **0.02** |
| TG (mmol/L) | -2.23 | 1.33 | 0.11 | 0.01 | 0.22 | 0.98 | -0.57 | 0.29 | **0.048** | -0.42 | | 0.36 | 0.24 | -1.27 | | 0.69 | 0.07 |
| Keratometry | -1.01 | 0.95 | 0.29 | -0.13 | 0.21 | 0.51 | -0.27 | 0.16 | **0.01** | -0.06 | | 0.19 | 0.75 | 0.14 | | 0.33 | 0.66 |
| BCVA (logMAR) | 10.58 | 4.62 | **0.034** | 0.37 | 1.15 | 0.75 | -3.26 | 1.00 | **0.001** | -1.40 | | 1.25 | 0.26 | -2.94 | | 2.10 | 0.16 |
| Cataract surgery |  |  |  | 1.32 | 3.17 | 0.68 |  |  |  | -0.49 | | 2.60 | 0.85 | -1.69 | | 2.13 | 0.43 |
| ACD (mm) | -1.35 | 1.75 | 0.45 | -0.21 | 0.52 | 0.69 | -0.66 | 0.47 | 0.16 | 0.22 | | 0.66 | 0.74 | -0.54 | | 1.14 | 0.64 |
| VCDR | -11.15 | 7.48 | 0.15 | -4.76 | 2.03 | **0.02** | -5.06 | 1.67 | **0.003** | -1.84 | | 2.16 | 0.39 | -6.05 | | 3.69 | 0.10 |
| CHD | 3.83 | 4.72 | 0.43 | -1.22 | 1.79 | 0.50 | 1.07 | 0.94 | 0.25 | -0.16 | | 0.98 | 0.87 | 0.07 | | 1.51 | 0.96 |
| IOP (mmHg) | 0.05 | 0.36 | 0.90 | -0.01 | 0.09 | 0.94 | -0.15 | 0.08 | 0.07 | 0.04 | | 0.09 | 0.68 | -0.28 | | 0.13 | **0.04** |
| SE (D) | 0.57 | 1.60 | 0.73 | 0.23 | 0.14 | 0.11 | -0.01 | 0.17 | 0.97 | -0.09 | | 0.18 | 0.61 | 0.38 | | 0.22 | 0.09 |
| AL (mm) | -0.06 | 0.85 | 0.94 | -0.22 | 0.21 | 0.29 | -0.09 | 0.20 | 0.64 | -0.08 | | 0.16 | 0.59 | -0.58 | | 0.46 | 0.21 |
| CCT (μm) | -0.03 | 0.03 | 0.43 | -0.01 | 0.01 | 0.19 | -0.01 | 0.01 | 0.35 | 0.01 | | 0.01 | 0.45 | 0.03 | | 0.02 | 0.05 |

| **Multivariate regression model** | | | | | | |  |  | | |  |  |  | | |  |  | |  |
| --- | --- | --- | --- | --- | --- | --- | --- | --- | --- | --- | --- | --- | --- | --- | --- | --- | --- | --- | --- |
| **Characteristic** | **≤40 years** | | |  | **41 - 49 years** | | |  | **50 - 59years** | | |  | **60 - 69 years** | | |  | **≥70 years** | | |
|  | **B** | **SE** | **P-value** |  | **B** | **SE** | **P-value** | | **B** | **SE** | **P-value** | | **B** | **SE** | **P-value** | | **B** | **SE** | **P-value** |
| Gender |  |  |  |  | 2.43 | 0.65 | **<0.001** |  |  |  |  |  |  |  |  |  |  |  |  |
| Current smoker |  |  |  |  |  |  |  |  |  |  |  |  | -2.01 | 0.73 | **0.006** |  |  |  |  |
| SBP (mm Hg) |  |  |  |  | -0.06 | 0.02 | **0.002** |  |  |  |  |  |  |  |  |  |  |  |  |
| Diabetes |  |  |  |  |  |  |  |  |  |  |  |  | 1.97 | 1.29 | 0.13 |  |  |  |  |
| LDL (mmol/L) | 4.45 | 1.29 | **0.002** |  |  |  |  |  |  |  |  |  |  |  |  |  | -1.57 | 0.70 | **0.03** |
| TG (mmol/L) |  |  |  |  |  |  |  |  | -0.62 | 0.27 | **0.029** |  |  |  |  |  | -1.05 | 0.69 | 0.13 |
| BCVA (logMAR) | 11.46 | 4.43 | **0.017** |  |  |  |  |  | -3.32 | 0.10 | **<0.001** |  |  |  |  |  |  |  |  |
| VCDR |  |  |  |  | -5.18 | 2.00 | **0.01** |  | -5.20 | 1.66 | **0.002** |  |  |  |  |  |  |  |  |

Note: Gender: male VS. female; Education: below high school VS. high school or above.

Supplementary Table 3. Demographic and biochemical factors in GCC thickness by AL groups.

**Univariate regression model**

| **Characteristic** | **<23 mm** | | | | **23–25 mm** | | | | **>25 mm** | | | |
| --- | --- | --- | --- | --- | --- | --- | --- | --- | --- | --- | --- | --- |
|  | **B** | **SE** | **t** | **P-value** | **B** | **SE** | **t** | **P-value** | **B** | **SE** | **t** | **P-value** |
| Age (years) | -0.19 | 0.02 | -8.23 | **<0.001** | -0.23 | 0.03 | -8.54 | **<0.001** | 0.22 | 0.22 | 1.01 | 0.33 |
| Gender | 0.79 | 0.43 | 1.83 | 0.07 | 0.85 | 0.51 | 1.66 | 0.10 | 1.75 | 4.61 | 0.38 | 0.71 |
| Education | 0.73 | 0.25 | 2.92 | **0.004** | 0.94 | 0.32 | 2.97 | **0.003** | 4.74 | 3.04 | 1.56 | 0.14 |
| Current smoker | -0.92 | 0.50 | -1.82 | 0.07 | -1.04 | 0.53 | -1.95 | 0.052 | -9.91 | 6.38 | -1.55 | 0.14 |
| BMI (kg/m2) | -0.02 | 0.06 | -0.39 | 0.70 | -0.02 | 0.07 | -0.32 | 0.75 | 0.06 | 0.58 | 0.10 | 0.92 |
| SBP (mm Hg) | -0.04 | 0.01 | -4.48 | **<0.001** | -0.05 | 0.01 | -4.35 | **<0.001** | 0.00 | 0.14 | -0.01 | 0.99 |
| DBP (mm Hg) | -0.03 | 0.02 | -1.61 | 0.11 | -0.02 | 0.02 | -1.16 | 0.24 | -0.09 | 0.16 | -1.25 | 0.57 |
| Diabetes | 2.72 | 1.06 | 2.58 | **0.01** | 3.94 | 1.43 | 2.75 | **0.006** |  |  |  |  |
| HbA1c (%) | -0.25 | 0.27 | -0.94 | 0.35 | -0.91 | 0.34 | -2.66 | **0.008** | -7.58 | 6.04 | -1.25 | 0.23 |
| HDL (mmol/L) | 0.74 | 0.78 | 0.96 | 0.34 | 0.09 | 0.92 | 0.09 | 0.93 | -2.29 | 7.21 | -0.32 | 0.76 |
| LDL (mmol/L) | -0.66 | 0.29 | -2.31 | **0.02** | -0.52 | 0.35 | -1.51 | 0.13 | -0.39 | 4.24 | -0.09 | 0.93 |
| TG (mmol/L) | -0.34 | 0.21 | -1.65 | 0.10 | -0.13 | 0.22 | -0.61 | 0.54 | -1.02 | 1.59 | -0.64 | 0.53 |
| Keratometry | -0.18 | 0.14 | -1.30 | 0.19 | -0.15 | 0.17 | -0.89 | 0.37 | -0.29 | 1.25 | -0.23 | 0.82 |
| BCVA (logMAR) | -3.69 | 0.74 | -5.00 | **<0.001** | -3.13 | 0.92 | -3.40 | **<0.001** | -6.94 | 5.70 | -1.22 | 0.24 |
| Cataract surgery | -2.15 | 2.10 | -1.02 | 0.31 | -3.24 | 2.13 | -1.52 | 0.13 | 5.83 | 9.29 | 0.63 | 0.54 |
| ACD (mm) | 0.07 | 0.49 | 0.15 | 0.88 | 0.47 | 0.53 | 0.89 | 0.38 | 4.13 | 0.01 | 3.24 | **<0.001** |
| VCDR | -4.08 | 1.46 | -2.79 | **0.005** | -5.38 | 1.68 | -3.21 | **0.001** | 6.40 | 0.03 | 2.54 | **<0.001** |
| CHD | 0.62 | 0.72 | 0.86 | 0.39 | 1.04 | 0.87 | 1.20 | 0.23 | -3.31 | 5.74 | -0.58 | 0.57 |
| IOP (mmHg) | -0.08 | 0.06 | -1.20 | 0.23 | 0.07 | 0.08 | 0.81 | 0.42 | -0.23 | 0.61 | -0.37 | 0.72 |
| SE (D) | 0.00 | 0.12 | 0.01 | 0.99 | -0.28 | 0.14 | -2.07 | **0.04** | -0.05 | 0.38 | -0.14 | 0.89 |
| CCT (μm) | 0.00 | 0.01 | -0.73 | 0.46 | 0.02 | 0.01 | 1.93 | 0.054 | -0.08 | 0.07 | -1.23 | 0.38 |

**Multivariate regression model**

| **Characteristic** | **<23 mm** | | | | **23–25 mm** | | | |
| --- | --- | --- | --- | --- | --- | --- | --- | --- |
|  | **B** | **SE** | **t** | **P-value** | **B** | **SE** | **t** | **P-value** |
| Age (years) | -0.177 | 0.025 | -6.930 | **<0.001** | -0.229 | 0.027 | -8.470 | **<0.001** |
| Gender | 1.195 | 0.425 | 2.810 | **0.005** | 0.726 | 0.640 | 1.20 | 0.23 |
| Current smoker |  |  |  |  | -0.797 | 0.629 | -1.27 | 0.205 |
| Diabetes | 1.961 | 1.028 | 1.910 | 0.057 | 2.432 | 1.380 | 1.76 | 0.078 |
| BCVA (logMAR) | -1.174 | 0.811 | -1.45 | 0.148 |  |  |  |  |
| VCDR | -4.428 | 1.426 | -3.10 | **0.002** | -5.220 | 1.617 | -3.230 | **0.001** |

Note: Gender: male VS. female; Education: below high school VS. high school or above.
